# Crosslinking and Swelling Properties of pH-Responsive Poly(Ethylene Glycol)/Poly(Acrylic Acid) Interpenetrating Polymer Network Hydrogels

**DOI:** 10.3390/polym16152149

**Published:** 2024-07-29

**Authors:** Uijung Hwang, HoYeon Moon, Junyoung Park, Hyun Wook Jung

**Affiliations:** Department of Chemical and Biological Engineering, Korea University, Seoul 02841, Republic of Korea; uj1023@grtrkr.korea.ac.kr (U.H.); hymoon@grtrkr.korea.ac.kr (H.M.); jypark@grtrkr.korea.ac.kr (J.P.)

**Keywords:** pH-responsive hydrogels, interpenetrating polymer networks, rheology, crosslinking, swelling, PEG, PAA

## Abstract

This study investigates the crosslinking dynamics and swelling properties of pH-responsive poly(ethylene glycol) (PEG)/poly(acrylic acid) (PAA) interpenetrating polymer network (IPN) hydrogels. These hydrogels feature denser crosslinked networks compared to PEG single network (SN) hydrogels. Fabrication involved a two-step UV curing process: First, forming PEG-SN hydrogels using poly(ethylene glycol) diacrylate (PEGDA) through UV-induced free radical polymerization and crosslinking reactions, then immersing them in PAA solutions with two different molar ratios of acrylic acid (AA) monomer and poly(ethylene glycol) dimethacrylate (PEGDMA) crosslinker. A subsequent UV curing step created PAA networks within the pre-fabricated PEG hydrogels. The incorporation of AA with ionizable functional groups imparted pH sensitivity to the hydrogels, allowing the swelling ratio to respond to environmental pH changes. Rheological analysis showed that PEG/PAA IPN hydrogels had a higher storage modulus (*G*′) than PEG-SN hydrogels, with PEG/PAA-IPN5 exhibiting the highest modulus. Thermal analysis via thermogravimetric analysis (TGA) and differential scanning calorimetry (DSC) indicated increased thermal stability for PEG/PAA-IPN5 compared to PEG/PAA-IPN1, due to higher crosslinking density from increased PEGDMA content. Consistent with the storage modulus trend, PEG/PAA-IPN hydrogels demonstrated superior mechanical properties compared to PEG-SN hydrogels. The tighter network structure led to reduced water uptake and a higher gel modulus in swollen IPN hydrogels, attributed to the increased density of active network strands. Below the p*K_a_* (4.3) of acrylic acid, hydrogen bonds between PEG and PAA chains caused the IPN hydrogels to contract. Above the p*Ka*, ionization of PAA chains induced electrostatic repulsion and osmotic forces, increasing water absorption. Adjusting the crosslinking density of the PAA network enabled fine-tuning of the IPN hydrogels’ properties, allowing comprehensive comparison of single network and IPN characteristics.

## 1. Introduction

Hydrogels are hydrophilic polymer networks with a three-dimensional structure, stabilized by crosslinking to prevent dissolution [1,2]. These materials have attracted considerable interest due to their potential applications in tissue engineering and drug delivery [3,4,5]. Capable of containing high water content, they promote biocompatibility. The porosity of hydrogels can be easily adjusted by manipulating the crosslinking density, allowing for the incorporation of drugs into the gel matrix [6,7]. Additionally, hydrogels exhibit several desirable characteristics for biomedical scaffold designs, such as support for cell migration and tissue integration, controlled release of growth factors, and tunable physical properties [8,9]. However, despite these promising attributes, the application of hydrogels is somewhat restricted by their inherently inferior mechanical properties [1,10,11].

Several strategies have been developed to enhance the mechanical properties of hydrogels. These strategies include increasing the crosslinking density, reducing the degree of swelling, and incorporating inorganic clays into the polymer to form nanocomposite gels [1,12]. Another effective approach involves incorporating interpenetrating network (IPN) systems, where two or more polymer networks are mutually independent but held together by internetwork entanglement. The concept of double networks (DN) was originally introduced by Gong et al. [13], who devised a sequential crosslinking method to create an IPN structure with remarkable mechanical properties, even in environments with high water content. In this arrangement, the initial rigid and brittle network serves as a sacrificial bond, while the subsequent soft and ductile network enables significant extensibility under stress [13,14]. Inspired by the DN concept, other hydrogel systems, such as bacterial cellulose/gelatin IPN gel [15], poly(ethylene glycol) (PEG)/poly(acrylic acid) (PAA) IPN gel [16], and hyaluronan/poly(N,N’-dimethylacrylamide) IPN gel [17] have been ingeniously developed. 

Myung et al. [16] developed a PEG/PAA IPN hydrogel system comprising a neutral crosslinked polymer in the primary network and an ionizable crosslinked polymer in the secondary network. This is the inverse of the IPN hydrogel system developed by Gong et al. [13]. PEG hydrogel is known for its excellent solubility in aqueous media, biocompatibility, and resistance to proteins and cells [18,19,20]. On the other hand, PAA is a pH-sensitive hydrophilic polymer highly compatible with living cells [21]. Its swelling behavior is greatly influenced by pH levels due to its ionizable and pH-sensitive carboxyl groups [22]. While the transparency, swelling, and mechanical properties of PEG/PAA IPN hydrogels have been examined as a function of pH [16,23,24,25,26,27,28], further research on their rheological crosslinking characteristics during IPN fabrication processes is still necessary, as it directly affects the final properties of the hydrogels.

Recent studies have focused on the rheological crosslinking characteristics and mechanical properties of photopolymerized PEG hydrogels under various conditions, including variations in the crosslinking ratio [29], solvent content [19], and the incorporation of silica nanoparticles [30]. Additionally, the relationship between rheological crosslinking features and curing conditions, such as UV irradiation time and temperature, has been explored [31]. To scrutinize the deeper relationship between crosslinking characteristics and the overall properties of PEG/PAA IPN hydrogels, rheological crosslinking and thermal properties were analyzed by altering the crosslinking density of the PAA networks. This analysis aimed to correlate these findings with swelling and mechanical properties under different pH conditions.

In this study, real-time rheological analysis was performed to explore the sequential free radical photopolymerization process involved in the fabrication of PEG/PAA IPN hydrogels. The incorporation of the PAA network into the PEG single network hydrogel led to the formation of hydrogen bonds between PEG and PAA below the p*K_a_* of acrylic acid (p*K_a_* = 4.3). The influence of the PAA network on the crosslinking density of PEG/PAA IPN hydrogels was studied by varying the ratio of acrylic acid (AA) monomer and poly(ethylene glycol) dimethacrylate (PEGDMA) crosslinker in the PEG/PAA IPN system. Rheological measurements were conducted under small-amplitude oscillatory shear (SAOS) mode during the photo-induced curing process of the PEG/PAA IPN hydrogels. To characterize the crosslinked networks, thermal properties were scrutinized using thermogravimetric analysis (TGA) and differential scanning calorimetry (DSC), and surface mechanical properties were assessed via nano-scratch tester (NST). Finally, the swelling and mechanical properties of the IPN hydrogels were evaluated in relation to pH levels.

## 2. Experimental Methods

### 2.1. Chemicals

**Figure 1:**
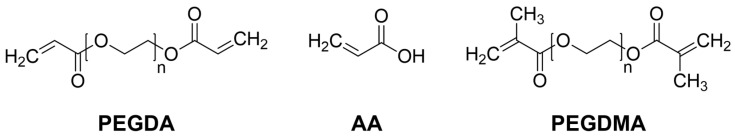
Chemical structures of PEGDA, AA, and PEGDMA.

2-Hydroxy-2-methylpropiophenone, acrylic acid (AA), poly(ethylene glycol) dimethacrylate (PEGDMA, Mw = 550), buffer solutions with pH values of 1, 4, and 7 were purchased from Sigma-Aldrich (St. Louis, MO, USA). Poly(ethylene glycol) diacrylate (PEGDA, Mw = 3400) was obtained from Alfa Aesar (Thermo Fisher Scientific Chemicals, Inc., Ward Hill, MA, USA). Chemical structures of PEGDA, AA, and PEGDMA are depicted in Figure 1.

### 2.2. Preparation of Hydrogels

#### 2.2.1. PEG Single Network Hydrogel (PEG-SN)

PEGDA was dissolved in deionized water (50 wt%) with 2-hydroxy-2-methylpropiophenone as a photoinitiator (1 wt% based on PEGDA). This solution formed a PEG single network (PEG-SN) through free radical photopolymerization induced by exposure to UV light (10 mW/cm^2^ for 180 s) [32].

#### 2.2.2. PEG/PAA IPN Hydrogels (PEG/PAA-IPNs)

**Figure 2:**
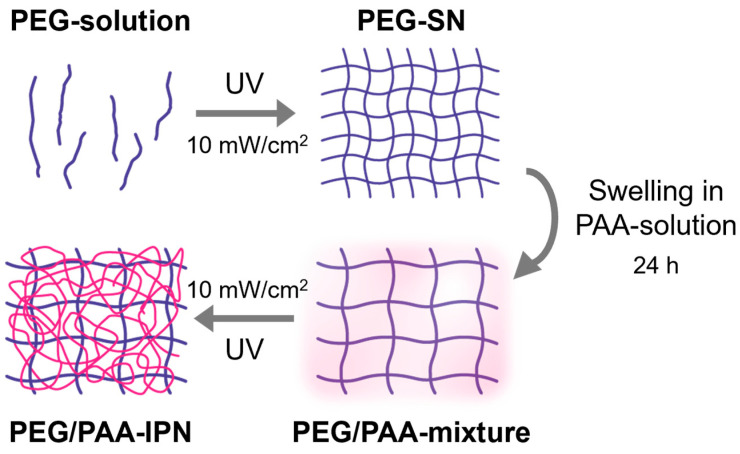
Fabrication process steps for PEG/PAA IPN hydrogels.

The aqueous acrylic acid solutions (PAA-solution) were prepared by dissolving acrylic acid in deionized water (50 vol%) with 2-hydroxy-2-methylpropiophenone as a photoinitiator (1 vol% based on acrylic acid) and PEGDMA as a crosslinker (1 and 5 vol% based on acrylic acid). PEG-SN was immersed in an aqueous acrylic acid solution for 24 h. After reaching its equilibrium swollen state, the PEG-SN swollen in PAA solutions with different portions of crosslinker were subsequently cured under the same UV conditions as the PEG-SN curing process. The fabricated IPN hydrogels were named PEG/PAA-IPN1 and PEG/PAA-IPN5, respectively. The formulations of the pre-polymerized solutions are detailed in Table 1, while the fabricating steps for the IPN hydrogels are outlined in Figure 2 [14]. 

### 2.3. Characterizations

#### 2.3.1. Raman Spectroscopy

The Raman spectra were acquired using a LabRam ARAMIS IR2 (HORIBA, Ltd., Kyoto, Japan) equipped with a 633 nm HeNe laser. The laser line was selected to minimize fluorescence interference. Laser power (17 mW at the sample) and acquisition time (120 s) were adjusted to improve the quality of the Raman spectra and prevent overheating. The crosslinking states of PEG-SN and PEG/PAA IPN hydrogels were confirmed by analyzing the changes in the Raman peaks before and after UV irradiation. 

#### 2.3.2. Thermogravimetric Analysis (TGA)

TGA measurements were conducted using a TGA 550 (TA Instruments, New Castle, DE, USA) to determine the thermal stability of the hydrogels. The analysis was performed from 30 °C to 600 °C at a heating rate of 10 °C/min under a constant nitrogen flow condition. The reported values from TGA measurements are the averages of three independent tests.

#### 2.3.3. Differential Scanning Calorimetry (DSC)

DSC experiments were carried out using an SDT Q600 DSC Q20 instrument (TA Instruments, New Castle, DE, USA) to measure the glass transition temperature of the hydrogels. This analysis was conducted from −50 °C to 150 °C at a heating rate of 10 °C/min under a constant nitrogen flow condition. The values of glass transition temperature (Tg) obtained are the averages of three independent tests.

#### 2.3.4. Rheological Measurements for UV Crosslinking of Hydrogels

An MCR-302 rotational rheometer (Anton Paar, Graz, Austria) equipped with an external UV light source (Omnicure 1500, Excelitas Technologies, Waltham, MA, USA) was utilized to monitor the real-time rheological properties during the photopolymerization of PEG-solution and PEG/PAA-mixtures. The UV light was emitted with a constant UV intensity of 10 mW/cm^2^ for 180 s from the quartz glass plate below. During the photopolymerization, a small amplitude oscillatory shear (SAOS) test was performed to measure the storage modulus (*G*′) at a constant strain of 0.1% and a constant angular frequency of 10 rad/s in the linear viscoelastic regime. The experiment employed an 8 mm diameter parallel-plate system, with test gaps of 500 μm and 700 μm for PEG-SN. Identical rheological data were obtained under both gap conditions. The crosslinking state of PEG/PAA IPN was assessed using an approximate 700 μm gap, corresponding to the equilibrium swelling of a 500 μm thick PEG-SN hydrogel in an aqueous acrylic acid solution, resulting in an approximate 700 μm thickness. Note that the final *G*′ values of hydrogels are the averages of three independent measurements. After fabricating the hydrogels using the described procedure, they were either dried at room temperature or immersed in varying pH solutions for approximately one week for further analysis. 

### 2.4. Swelling Ratio

Swelling ratios of PEG-SN and PEG/PAA-IPN hydrogels were measured at different pH values. The hydrogel films produced via the rheology tests were immersed in buffer solution with pH values of 1, 4, and 7, respectively, for at least one week. Subsequently, the hydrogels were removed from the buffer solution, and their weights were measured after excess solution was removed from their surfaces. The hydrogels were then dried in an oven at 60 °C for 24 h to achieve a fully dried state. The swelling ratio (*Q*) was calculated using the following equation:

(1) $$Q=\frac{w_{s}-w_{d}}{w_{d}}$$

where, *w_s_* and *w_d_* represent the weights of the hydrogel in a fully swollen state and fully dried state, respectively [33]. The swelling tests were conducted in triplicate.

### 2.5. Mechanical Tests

#### 2.5.1. Nano-Scratch Tests

The surface mechanical resistance of PEG-SN and PEG/PAA-IPNs in a dried state was assessed using a nano-scratch tester (NST^3^, Anton Paar Tritec SA, Peseux, Switzerland). The scratch procedure involves three sequential scans: First, an initial surface probing (pre-scan) under a constant load of 0.1 mN; second, scratching the hydrogel surface with a diamond tip in progressive normal force mode (ranging from 0.1 to 10 mN at a rate of 19.8 mN/min); third, a post-scan under 0.1 mN to measure the residual depth after the scratching test. The penetration depth and residual depth were accurately quantified over the total length (1 mm) of the scratch. A three-dimensional image of the scratched area was captured through AFM (AFM Wide Scan, Anton Paar Tritec, Peseux, Switzerland) attached to the NST equipment at a position 0.5 mm from the starting point of the scratch pattern [19,29]. 

#### 2.5.2. Measurements of Shear Storage Modulus (G′) and Compression Modulus (E)

The shear storage modulus (*G*′) and compression modulus (*E*) of the equilibrium-swollen PEG-SN and PEG/PAA-IPN hydrogels were determined using an MCR 302 rheometer. These hydrogels, prepared with an 8 mm diameter and a thickness of approximately 1 mm, were exposed to buffer solutions with pH values of 1, 4, and 7. To prevent slippage during the tests, sandpaper was placed on both the upper and lower plates of the rheometer. Compression tests were conducted at a strain rate of 0.2 mm/min and were stopped once the normal force reached 50 N. The compression modulus was calculated using the stress-strain data collected between 5% and 10% strain. For the oscillatory frequency sweep tests, a parallel-plate system with an 8 mm diameter was utilized, and the gap was adjusted until the normal force reached 0.05 N. Tests were performed at a strain of 0.01% within the linear viscoelastic regime, and the shear storage modulus at an angular frequency of 0.1 rad/s was analyzed. The compression and oscillatory frequency sweep tests were conducted in triplicate, respectively.

## 3. Results and Discussion

### 3.1. Variation of C=C Double Bonds during UV Irradiation

**Figure 3:**
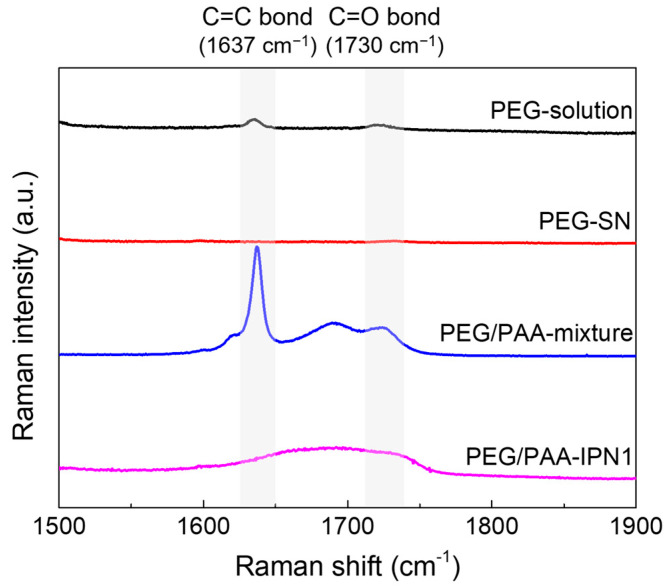
Raman spectra of hydrogels before and after UV irradiation during the fabrication of PEG/PAA IPN hydrogels.

Raman spectra were acquired before and after UV exposure during the fabrication process of PEG-SN and PEG/PAA-IPN1 hydrogels, as depicted in Figure 3. To enhance spectral resolution, PEG-solution was replaced with PEGDA powder. After UV exposure, the disappearance of the C=C peak at 1637 cm^−1^ [19,29,31] in the PEG-solution indicated the progress of free radical polymerization. Subsequently, the reappearance of the C=C peak in the PEG/PAA-mixture state was attributed to the penetration of the PAA-solution (containing both AA and PEGDMA with C=C double bonds) within the PEG-SN hydrogel framework. Furthermore, a reduced C=C peak in the PEG/PAA-IPN1 after UV exposure supported the formation of the PAA network, which independently intermingled with the PEG network through photopolymerization. Additionally, the presence of the C=O peak, typically observed at approximately 1722 cm^−1^ [31], might have been influenced by the C=C peak Raman shift within the samples of PEG/PAA-mixture and PEG/PAA-IPN1. Both PEG-SN and PEG/PAA-IPN1 hydrogels were measured in the same dried state, enabling a direct comparison of their Raman spectra peaks. Despite the presence of C=O bonds in PEG-SN hydrogels, minimal intensity around 1730 cm^−1^ was observed compared to the PEG/PAA-IPN1 hydrogels. This difference likely arose from variations in C=O bond concentration, influenced by the higher molecular weight of PEGDA compared to AA. 

### 3.2. Real-Time Crosslinking Behavior during UV Irradiation

**Figure 4:**
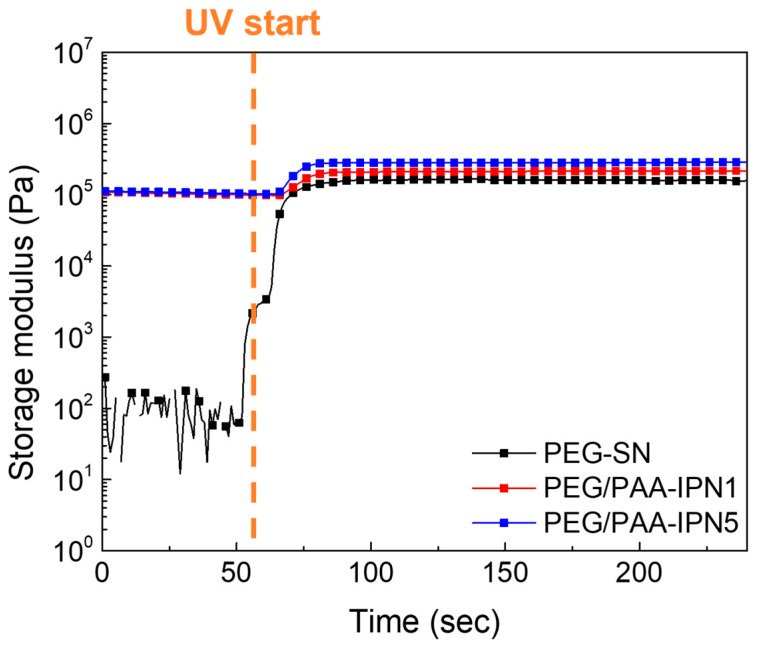
Real-time curing behaviors of PEG-SN, PEG/PAA-IPN1, and PEG/PAA-IPN5.

The chemo-rheological evolutions of PEG-SN and PEG/PAA-IPNs during photopolymerization were monitored by measuring the *G*′ in real time, as shown in Figure 4. The abrupt increase in the *G*′ value of the PEG-solution upon UV irradiation reflects the development of a 3D crosslinked network through the initiation of free radical polymerization and radical crosslinking reactions, converting C=C bonds in acrylate groups into C–C bonds within the hydrogels [30,31]. It should be noted that the initial *G*′ before UV irradiation of PEG/PAA-mixtures was lower than the plateau *G*′ (final *G*′) of PEG-SN because PEG-SN was immersed in the PAA-solution prior to the secondary curing process. Subsequent UV irradiation led to an increase in *G*′, indicating additional polymerization of the PAA-solution within the PEG-SN matrix. The correlation between rheological and Raman spectroscopy results substantiates the formation of the IPN structure. Considering the rheological crosslinking behaviors of PEG/PAA-IPN1 and PEG/PAA-IPN5, their *G*′ values were almost similar initially. However, after UV irradiation, the final *G*′ values of PEG/PAA-IPN1 and PEG/PAA-IPN5 were measured as (2.15 ± 0.03) × 10^5^ and (2.87 ± 0.02) × 10^5^ Pa, respectively. This disparity underscores the formation of a denser crosslinked network in PEG/PAA-IPN5, attributed to the higher concentration of crosslinker within the PAA-solution [19,29]. 

### 3.3. Thermal Properties of PEG-SN and PEG/PAA-IPNs

**Figure 5:**
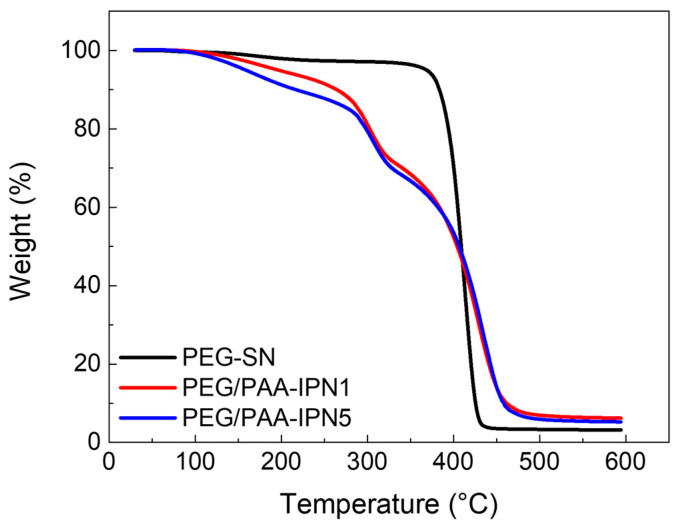
TGA thermograms of PEG-SN, PEG/PAA-IPN1, and PEG/PAA-IPN5.

The decomposition and thermal stability of the fabricated hydrogels were investigated using TGA, as illustrated in the weight loss curves in Figure 5. PEG-SN exhibited a single sharp weight loss step, whereas PEG/PAA-IPN1 and PEG/PAA-IPN5 showed two distinct weight loss regions. The maximum peak temperature (T_max_) of the degradation steps was determined based on the maximum peak of the derivative of weight change with respect to temperature for each weight loss region [34]. The T_max_ values for PEG-SN and PEG/PAA-IPN hydrogels are listed in Table 2. The T_max_ values at the second degradation step for PEG/PAA-IPN1 and PEG/PAA-IPN5 were found to be somewhat similar to that at the single degradation step for PEG-SN, suggesting that the second degradation step of PEG/PAA-IPNs is closely linked to the degradation of the crosslinked PEG network. Thus, the first degradation step of PEG/PAA-IPNs is primarily related to the degradation of the crosslinked PAA network. Importantly, the T_max_ of the first degradation step for PEG/PAA-IPN5 was higher than that of PEG/PAA-IPN1, implying that a higher concentration of crosslinker in the PAA-mixture improved the thermal stability of the crosslinked PAA network. T_max_ at the second decomposition step for PEG/PAA-IPNs was also slightly higher than the degradation temperature of PEG-SN, indicating that the supplementary IPN structure contributed to the improved thermal stability of the crosslinked PEG network [35]. 

**Figure 6:**
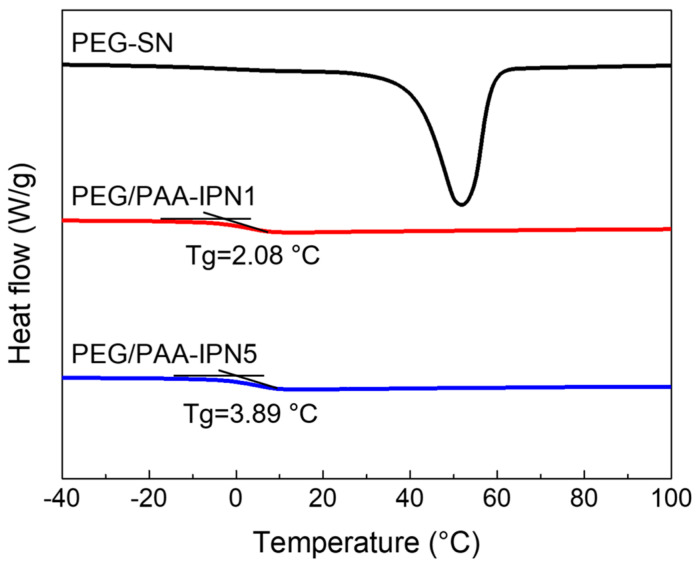
DSC thermograms of PEG-SN, PEG/PAA-IPN1, and PEG/PAA-IPN5.

The thermal changes during the heating regime of both PEG-SN and PEG/PAA-IPNs were evaluated using DSC (Figure 6). PEG-SN was not included in the comparison due to difficulties in determining its Tg, while the Tg values for PEG/PAA-IPN1 and PEG/PAA-IPN5 were recorded as 2.08 ± 0.27 °C and 3.89 ± 0.30 °C, respectively. Despite being thermosets, the presence of a melting temperature suggests that some crystalline segments inside the hydrogel might undergo melting, even though the hydrogel as a whole did not melt [36]. The Tg of PEG/PAA-IPN5 was slightly higher than that of PEG/PAA-IPN1, due to the increased crosslinking density of the PAA network [37].

### 3.4. Surface Mechanical Properties of PEG-SN and PEG/PAA-IPNs

**Figure 7:**
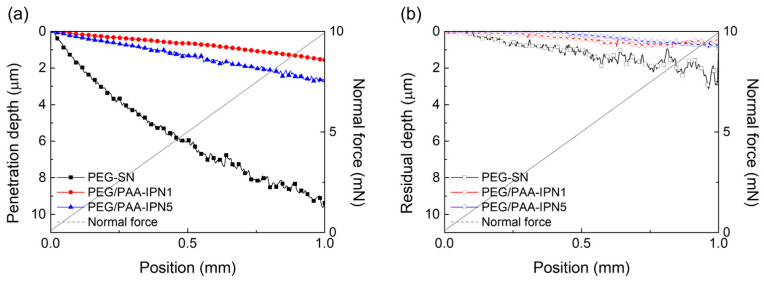
(**a**) Penetration depth and (**b**) residual depth profiles of PEG-SN, PEG/PAA-IPN1, and PEG/PAA-IPN5 obtained by NST.

**Figure 8:**
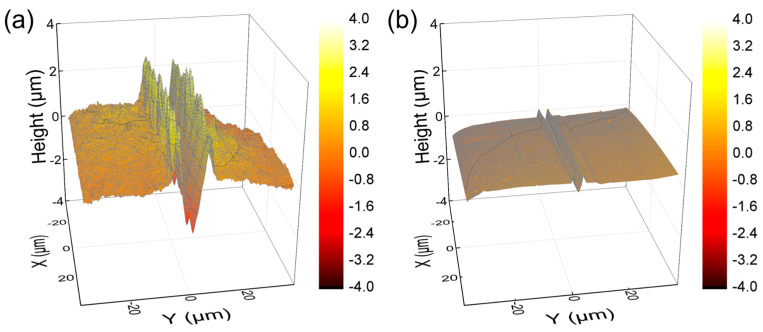
AFM images of residual depth profiles of (**a**) PEG-SN and (**b**) PEG/PAA-IPN1 at a 0.5 mm scanned position.

The surface mechanical properties of hydrogel films were evaluated using NST to investigate scratch resistance by measuring penetration and residual depths. Hydrogel films with less densely crosslinked structures generally exhibited deeper scratch profiles [31,38,39]. As shown in Figure 7, PEG-SN had a significantly deeper and more uneven penetration depth compared to PEG/PAA-IPNs, confirming the presence of a looser crosslinked network. The second curing process involved in forming the IPN from PEG-SN to PEG/PAA-IPNs resulted in a tighter network structure. PEG/PAA-IPN5 demonstrated the shallowest penetration depth due to enhanced mechanical hardness stemming from a higher concentration of crosslinker in the PAA network. PEG-SN displayed a greater residual depth compared to PEG/PAA-IPNs, with no significant difference observed in residual depths among the PEG/PAA-IPN hydrogels. In addition, the deeper and more fluctuated residual depth for PEG-SN was also observed in the AFM images, unlike PEG/PAA-IPN1 (Figure 8). Note that PEG/PAA-IPN1 and PEG/PAA-IPN5 exhibited similar AFM profiles.

### 3.5. pH-Dependent Swelling and Mechanical Properties

**Figure 9:**
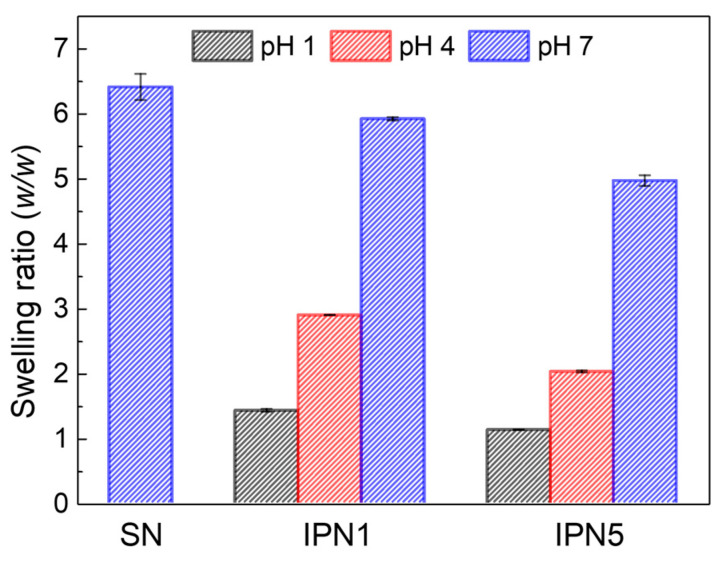
Swelling ratios as a function of pH for PEG-SN, PEG/PAA-IPN1, and PEG/PAA-IPN5.

The swelling ratios of PEG-SN, PEG/PAA-IPN1, and PEG/PAA-IPN5 hydrogels were measured as a function of pH. The swelling ratio of PEG-SN, which remained constant regardless of pH, is presented at pH 7 (Figure 9). In contrast, the swelling ratio of the IPN hydrogels increased with rising pH levels, demonstrating the tunable swelling feature of PEG/PAA IPN hydrogels according to pH. At pH 7, the swelling ratio was highest for PEG-SN, followed by PEG/PAA-IPN1, and lowest for PEG/PAA-IPN5. This result directly supports the observation that a hydrogel with a denser crosslinked network exhibits a lower swelling ratio. At pH levels above the p*Ka* of acrylic acid (4.3), the ionization of carboxyl groups enhances both the ionic osmotic pressure and the electrostatic repulsion, leading to a significant increase in swelling [5,22]. Furthermore, at pH levels below the p*Ka* of acrylic acid, PEG and PAA form a complex between independent networks through hydrogen bonding interactions [16,26]. This pH-dependent swelling behavior is attributed to the polyelectrolyte nature of the PAA network [11]. 

**Figure 10:**
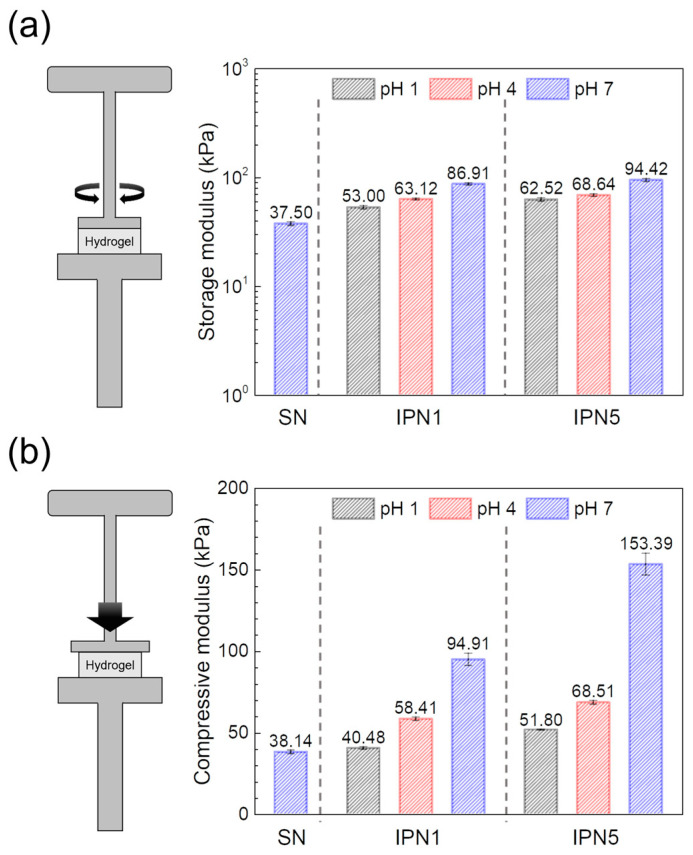
Moduli obtained from (**a**) shear tests and (**b**) compressive tests of swollen hydrogels at different pH levels.

Contrary to the observed trends in swelling ratio behavior, the IPN hydrogels exhibited significantly higher storage and compressive moduli compared to the PEG-SN hydrogels. This enhancement in mechanical properties suggests a more robust network structure within the IPN hydrogels. Moreover, the moduli of PEG/PAA-IPN5 surpassed those of PEG/PAA-IPN1 under the same pH conditions, indicating that the degree of crosslinking in network strands [29,40], a dominant factor affecting modulus, contributes to superior mechanical properties (Figure 10). In the structure of IPN hydrogels, an increase in swelling ratio typically corresponds to a reduced concentration of elastically active network chains, which generally leads to a decrease in the gel modulus [27]. However, contrary to this trend, the gel modulus increased with the rise in pH, despite the increased swelling ratio and reduced concentration of elastically active network chains. A similar observation was reported by Waters et al. [26] in their study on the compressive modulus of PEG/PAA IPN hydrogels as a function of pH. In PEG/PAA IPN hydrogels, an increase in pH leads to the ionization of the PAA networks, resulting in significant swelling due to additional electrostatic repulsion and osmotic forces [41]. The PAA networks are forced to expand substantially as they intermingle with the PEG network within IPN structure. This expansion, approaching the limits of finite chain extensibility, increases the modulus due to the substantial entropic penalty associated with stretching network chains close to full extension [26,42]. Consequently, the moduli of PEG/PAA IPN hydrogels increase concurrently with rising pH levels, consistent with their swelling behaviors. 

## 4. Conclusions

In this study, we incorporated PAA networks into PEG networks to develop IPN hydrogel systems and performed various tests to assess crosslinking properties and pH-dependent swelling and mechanical properties. To fabricate the PEG/PAA IPN hydrogels, the PEG-SN hydrogels were first produced through a UV curing process. Subsequently, the PEG-SN hydrogels were immersed in PAA-solutions with different molar ratios of AA monomer and PEGDMA crosslinker, and exposed to secondary UV irradiation to form independent PAA networks within the primary PEG networks. Through these fabrication processes, the formation of PEG/PAA IPN hydrogels, comprising two individually crosslinked networks, was confirmed by the variation of C=C double bonds using Raman spectroscopy. Specifically, real-time rheological measurements indicated that the PEG/PAA-IPN hydrogels had more compact networks compared to PEG-SN hydrogels, as evidenced by the plateau *G*′ values observed during the UV curing process. Moreover, PEG/PAA-IPN5 exhibited a denser structure and higher thermal stability than PEG/PAA-IPN1, indicating a higher crosslinked network density due to the increased crosslinker content within the PAA networks. For mechanical properties, PEG/PAA-IPN hydrogels displayed a denser network structure, which influenced their swelling properties, resulting in lower water content but higher gel moduli compared to PEG-SN hydrogels. The enhanced gel modulus in PEG/PAA-IPN5 was attributed to a higher concentration of elastically active network strands and increased crosslinking density. The PAA network introduced unique pH-responsive properties to the IPN hydrogels; below the pKa of acrylic acid, hydrogen bonding between PEG and PAA networks led to deswelling, while above the pKa, electrostatic repulsion and osmotic forces increased the water content in hydrogels. Furthermore, the modulus of the IPN hydrogels increased with rising pH, primarily due to the finite extensibility of the PEG network chains. Overall, the real-time crosslinking behavior and structural properties of the PEG/PAA IPN hydrogels corroborated their swelling and mechanical characteristics. By precisely adjusting the crosslinking density of the PAA network, we demonstrated the capability to fine-tune the hydrogel properties, facilitating a comprehensive comparison between PEG single network and IPN configurations. The synergistic attributes of this intricately IPN-structured hydrogel system, crafted through a UV curing process, underscore its potential for various biomedical applications.

## Figures and Tables

**Table 1:** Formulations of pre-polymerized solutions for PEG/PAA IPN hydrogels.

Sample	Weight Fraction (wt%)				
	PEGDA	AA	PEGDMA	DI Water	PI
PEG-solution	49.75	-	-	49.75	0.5
PAA-solution 1	-	50.676	0.536	48.263	0.525
PAA-solution 5	-	49.558	2.730	47.198	0.513

**Table 2:** The maximum peak temperatures of the first (T_max,1_) and second (T_max,2_) degradation steps obtained from TGA tests for PEG-SN and PEG/PAA-IPN hydrogels.

Hydrogels	T_max,1_ (°C)	T_max,2_ (°C)
PEG-SN	414.16 ± 1.00	-
PEG/PAA-IPN1	301.85 ± 2.11	432.14 ± 0.99
PEG/PAA-IPN5	307.13 ± 0.76	431.37 ± 2.39

## Data Availability

The original contributions presented in the study are included in the article, further inquiries can be directed to the corresponding author.
